# CACP syndrome (Camptodactyly Arthropathy Coxa Vara Pericarditis). Clinical case

**DOI:** 10.1186/1546-0096-9-S1-P215

**Published:** 2011-09-14

**Authors:** E Košková, E Vrtíková

**Affiliations:** 1 National Institute of Rheumatic disease, Pieštany, Slovak Republic

## Introduction

The CACP syndrome is characterized by congenital camptodactyly and early childhood onset of non inflammatory synovial hyperplasia. Some patients present with coxa vara, others with pericarditis, some have both of these symptoms.

## Case Report

Our patient, a 6 year old girl was diagnosed with the CACP syndrome at the age of 30 months. Her parents were negative for consanguinity. As an infant the patient was diagnosed with camptodactyly. At the age of 18 months frequent swelling of the knee was observed. Examination by rheumatologist confirmed the presence of swelling to elbows, wrists, knees, and ankles, without limitation of range of motion. Markers of inflammation were negative, inflammation was excluded also by analysis of the synovial fluid. Ultrasonography showed noninflammatory synovial hyperplasia as well as accumulation of the intraarticular fluid.

Radiology exams of the hip joints revealed early signs of dysplasia.

At the age of 6 years the X-ray of pelvis confirmed the presence of the coxa vara.

The syndrome is currently managed with intensive physiotherapy.

## Conclusion

1. The CACP syndrome may be diagnosed mistakenly as a juvenile idiopatic arthritis.

2. Ultrasonography as the method of examination seems to be very important for the diagnosis of the CACP syndrome. In our patient the findings of ultrasonography, together with the history of camptodactyly in infancy and negative markers of inflammation were crucial for the differential diagnosis.

3. Over the following years coxa vara developed in our patient. This pathology is another diagnostically important symptom of the CACP syndrome.

